# Supplementary figures and images for: UM171 induces a homeostatic inflammatory-detoxification response supporting human HSC self-renewal
Source: PLoS One. 2019 Nov 8;14(11):e0224900. doi: 10.1371/journal.pone.0224900 (PMC6839847; doi:10.1371/journal.pone.0224900)

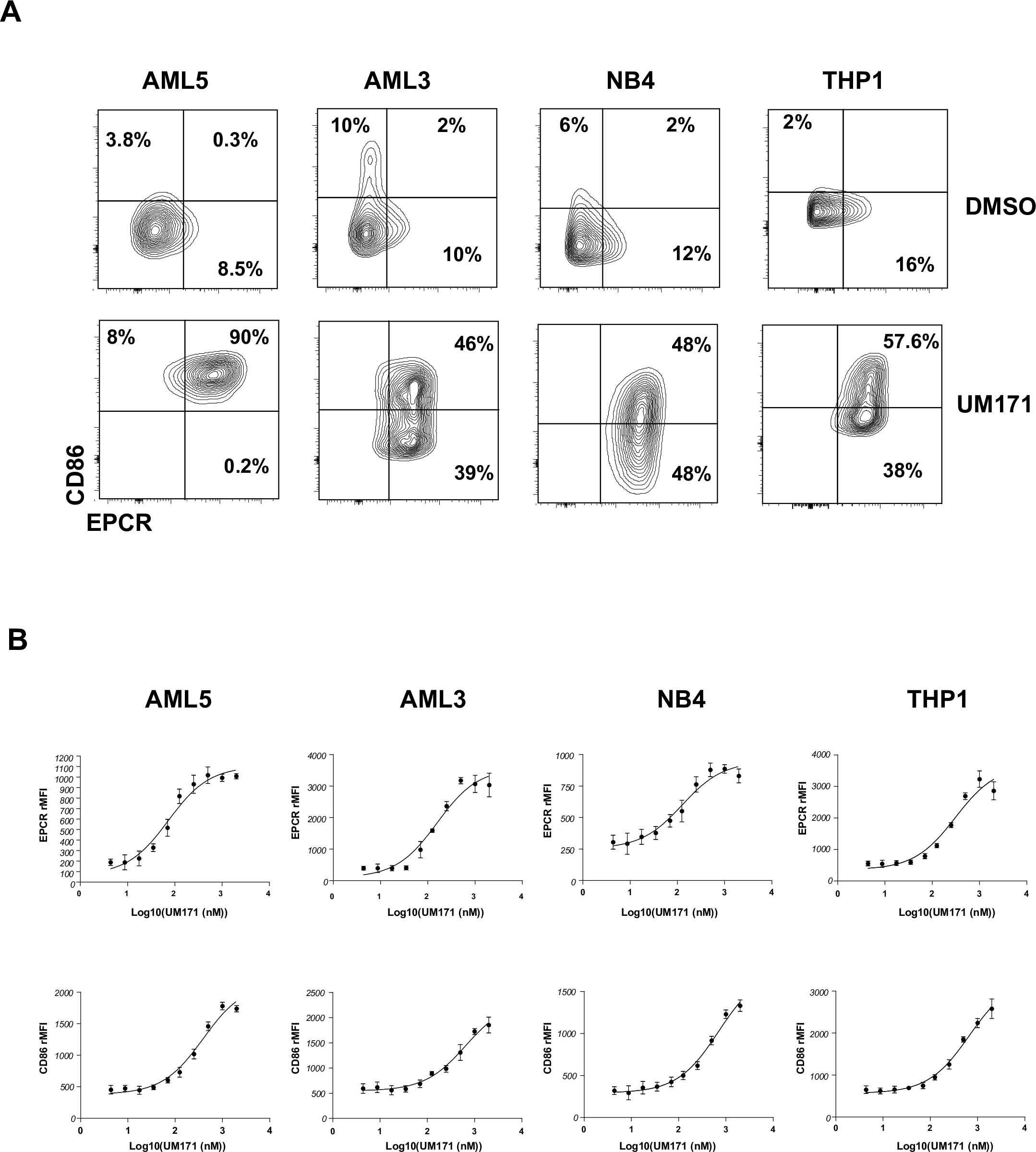

Supplement: S1 Fig — A: Representative FACS profiles of CD86 and EPCR co-expression in monocytic derived cell lines exposed to DMSO or UM171 (500nM) for 24h. Various myeloid derived cell lines were screened for the upregulation of both EPCR and CD86 in response to UM171. Among them, acute myeloid leukemia cell lines OCI-AML3 and OCI-AML5 (FAB M4), promyelocytic leukemia NB4 (FAB M3) and monoblastic leukemia THP-1 (FAB M5) are shown. Note that OCI-AML5 cell line was used for all further studies as it shows the most consistent and highest response to UM171. B: Dose response curves for UM171-induced EPCR (upper panel) and CD86 (lower panel) expression in each cell lines (relative mean fluorescence intensity was assessed by flow cytometry). Data are expressed as mean ± SEM of 3 independent experiments. (TIF) [file pone.0224900.s001.tif]

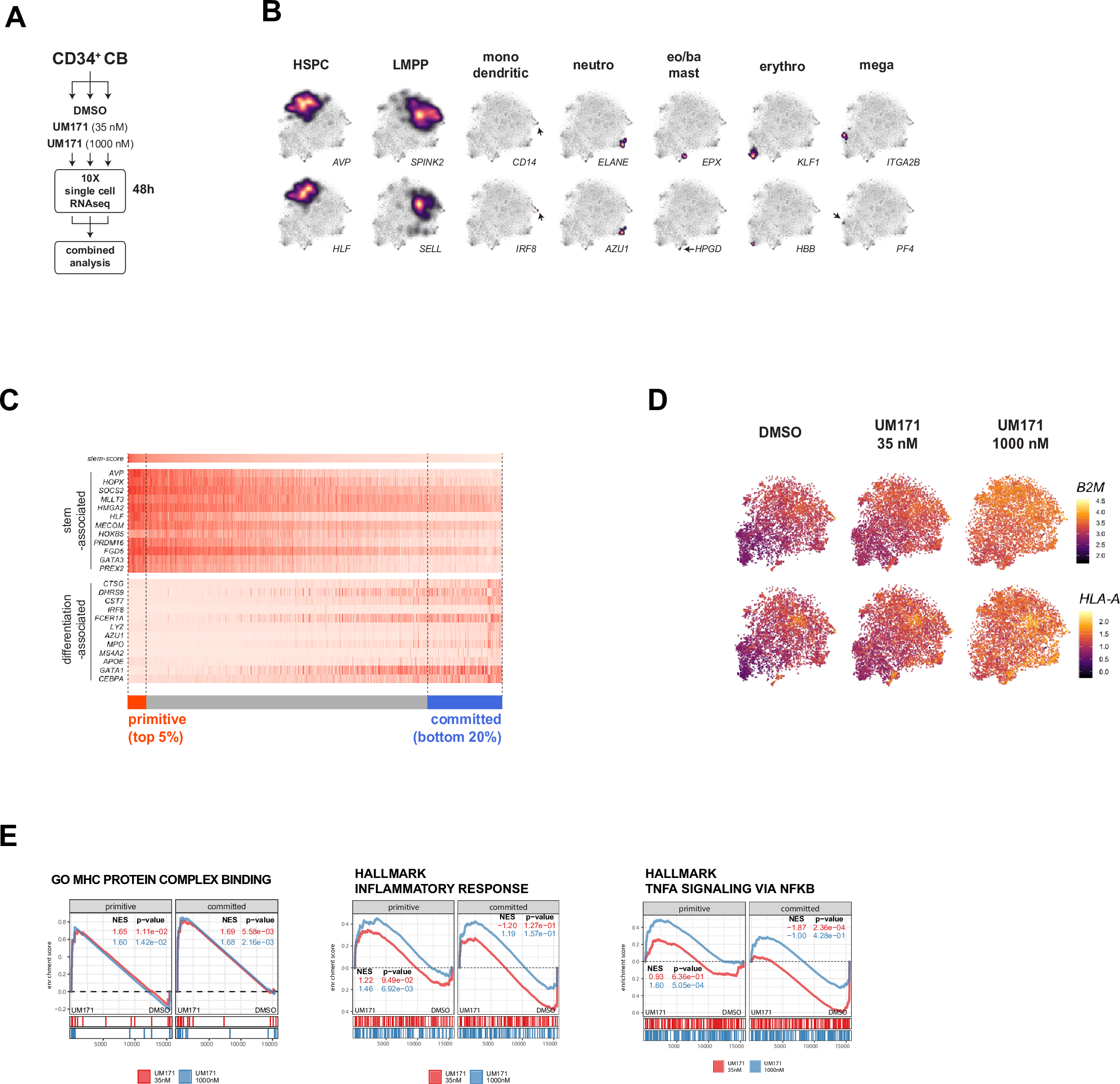

Supplement: S2 Fig — A: Experimental design to identify UM171 induced transcriptomic changes in single CD34+ cord blood cells. B: Combined t-SNE projections (grey dots) of a total of 16,669 CD34+ CB cells treated with either DMSO or two different doses of UM171 (35 and 1000 nM). Cell populations were identified by key marker expression and are plotted on top of t-SNE map. HSPC: hematopoietic stem and progenitor cells; LMPP: lymphoid primed multi-potent progenitors; mono/dendritic: mature monocytic/dendritic cells; neutro: neutrophils, eo/ba/mast: eosinophils/basophils/mast cells; erythro: erythoid cells; mega: megakaryocytic cells. Cellular phenotypes in the central t-SNE projection space exhibited less discrete but more transitionary gene expression patterns (not shown), consistent with intermediate differentiation states and progressive lineage specification. C: Heatmap of stem cell associated genes across 16,669 cells used for calculation of a stem score, and selected differentiation genes. Bar plot (bottom) represents the cutoff for categorization into primitive and committed cell subsets. D: t-SNE heatmap of representative inflammatory genes B2M and HLA-A; imputed data (MAGIC). E: GSEA enrichment of selected inflammation associated genesets. (TIF) [file pone.0224900.s002.tif]

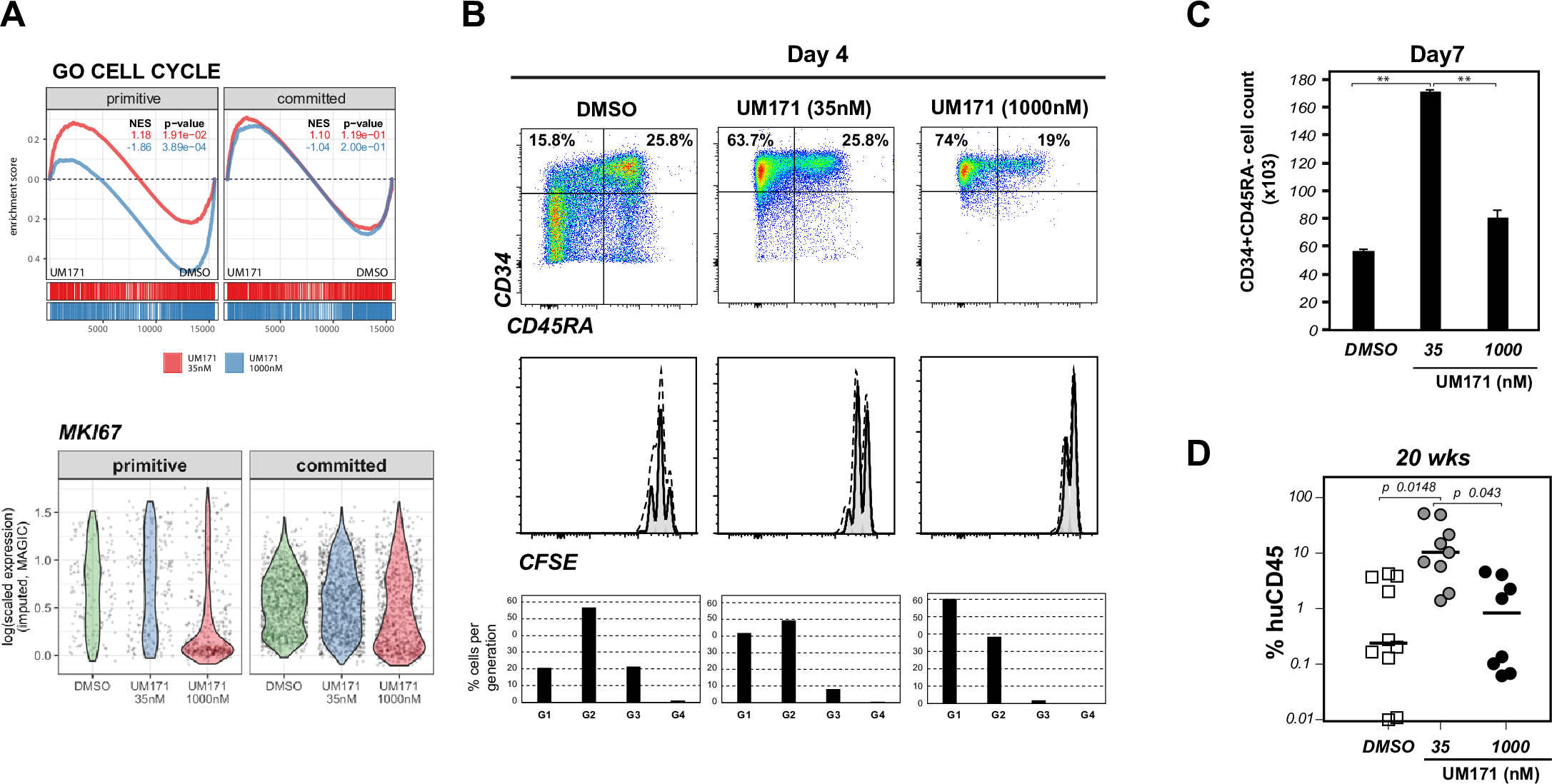

Supplement: S3 Fig — A: GSEA enrichment summary indicating a selective cell cycle blockade in the primitive cell subset treated with 1000 nM UM171 (upper panel). Violin plots of distributions of expression levels of cell cycle gene MKI67 (lower panel). Note the selective reduction of MKI67-expressing cells in primitive UM171 (1000nM) treated subset (imputed single cell expression data). B: CD34+ cord blood cells were cultured for 4 days in presence of DMSO or UM171 (35nM and 1000nM). Percentage of CD34+CD45RA- HSC enriched subset are shown in upper panel. Cell division of CD34+CD45RA- subsets was assessed using CFSE staining method (lower panel). Graph show % of cells in each generation. C: CD34+ cord blood cells were cultured for 7 days in presence of DMSO or UM171 (35nM and 1000nM). CD34+CD45RA- enriched HSC cell count were assessed before transplantation. D: Day 7 cultures exposed to DMSO or UM171 (35nM and 1000nM) were transplanted in immunocompromised NSG mice (outcome of 2 CRU). Human CD45 engraftment was assessed at 20 wks post-transplantation. Note that high dose of UM171 affect its capacity to expand HSCs with long-term repopulating activity. (TIF) [file pone.0224900.s003.tif]

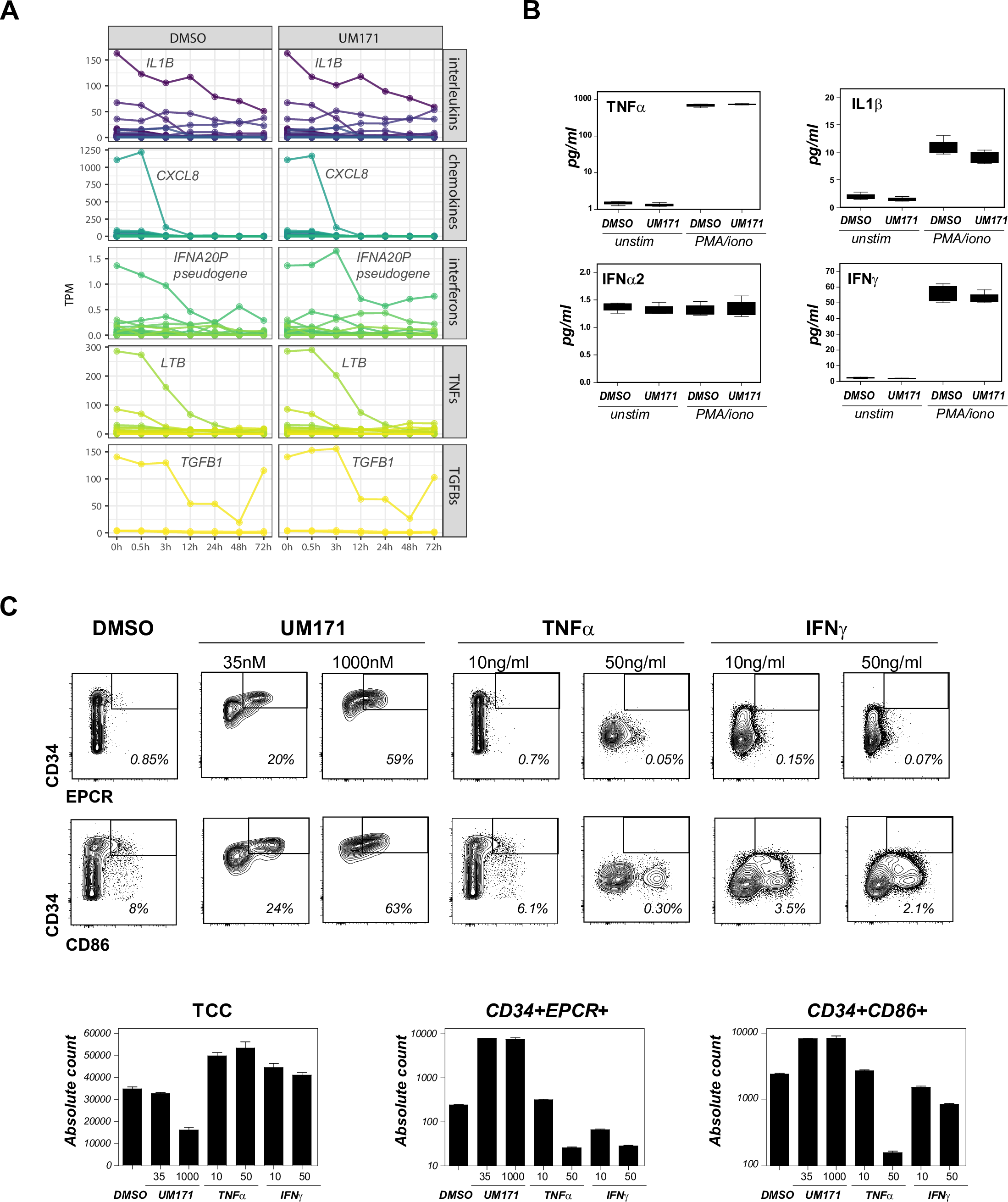

Supplement: S4 Fig — A: Expression trajectories of interleukin, chemokine, interferon, TNF and TGFb family members in DMSO versus UM171 (35nM) treated CD34+ cord blood cells. Gene family annotations were downloaded from HUGO gene nomenclature committee (www.genenames.org). B: Amounts of pro-inflammatory cytokines IL1b, TNFa, IFNa2 and IFNg were measured by flow cytometry (LegendPlex) in day4 DMSO or UM171 exposed CD34+ culture media. Note that secretion of these pro-inflamatory cytokines were not induced by UM171 even after PMA/ionomycin stimulation. C: CD34+ cord blood cells were cultured for 4 days in presence of DMSO or UM171 (35 and 1000nM), or pro-inflammatory cytokine TNFa (10 and 50ng/ml) or IFNg (10 and 50ng/ml). CD34, EPCR and CD86 surface expression were assessed by flow cytometry. Representative FACS profile (upper panels) showing % of CD34+EPCR+ and CD34+CD86+ subsets and absolute counts (lower panels) of indicated populations in each condition.\ (TIF) [file pone.0224900.s004.tif]

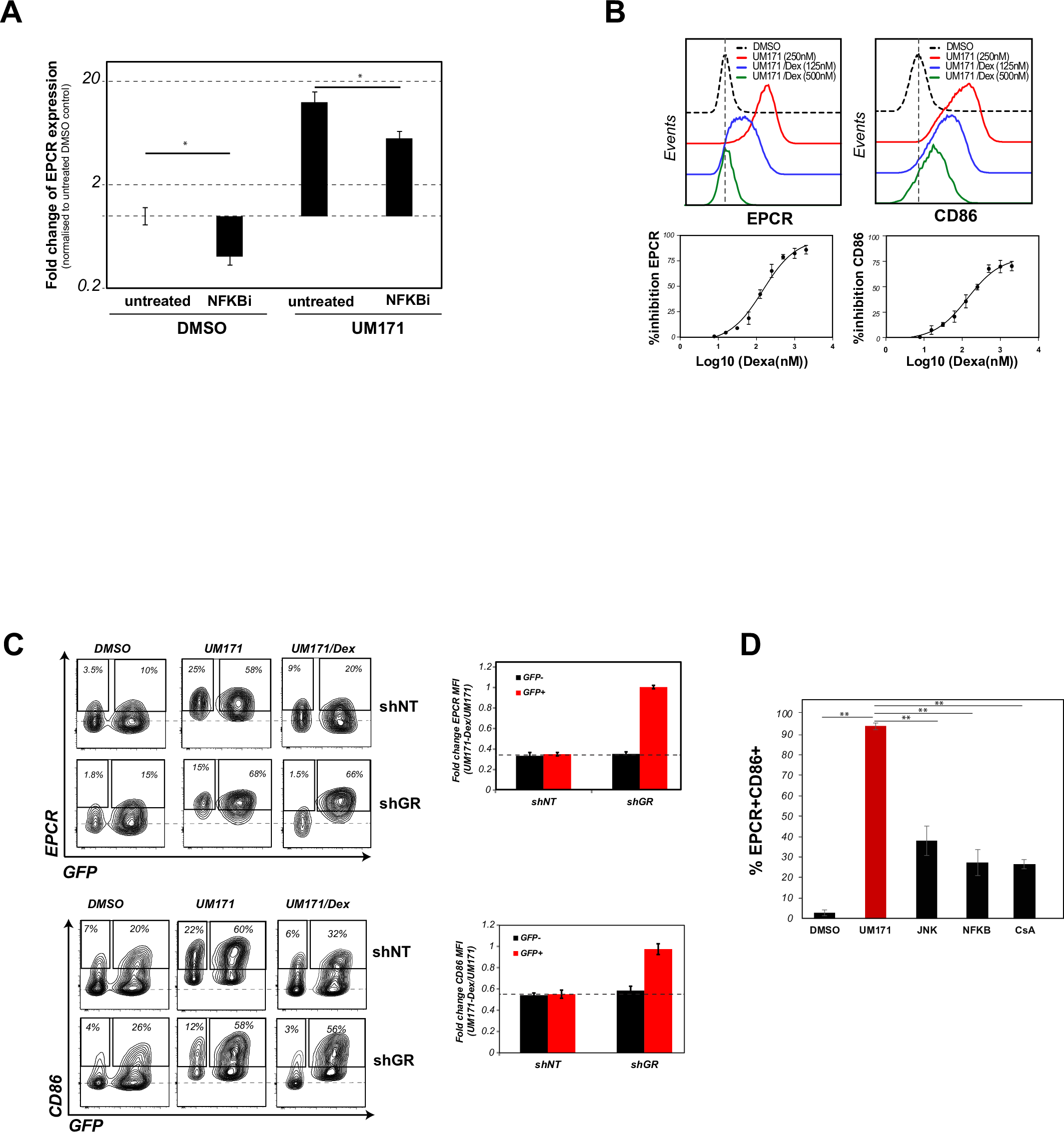

Supplement: S5 Fig — A: Modulation of EPCR mRNA levels in response to NFKB inhibitor in enriched HSC subset. Data shown represent mean fold change in EPCR expression (± S.E.M.) of sorted CD34+CD45RA- cells cultured for 48h in presence of DMSO, UM171 (35nM), NFKB inhibitor (EVP4593, 100nM) and UM171 + EVP4593 (representative of 2 independent specimen done in quadruplicates). B: Representative FACS profile (upper panel) and inhibition curves (lower panel) of UM171 mediated EPCR and CD86 induction after dexamethasone treatment. Data are shown as mean ± SEM for 2 independent experiments. C: GR knockdown was performed in OCI-AML5 cells. Transduced cells were exposed to DMSO, UM171 (250nM) or UM171 (250nM)/Dex (100nM) and EPCR and CD86 expression were evaluated by flow cytometry. Representative FACS profiles are shown in left panel and inhibition response to dexamethasone are presented in right panel. Data are shown as mean ± SEM for 2 independent experiments. D: Percentage of UM171 induced CD86+ EPCR+ OCI-AML5 cells after 2 days treatment with following immunosuppressive drugs: (JNK inhibitor (SP600125, 200nM), NFKB inhibitor (EVP4593, 100nM), cyclosporine A (10nM). (TIF) [file pone.0224900.s005.tif]

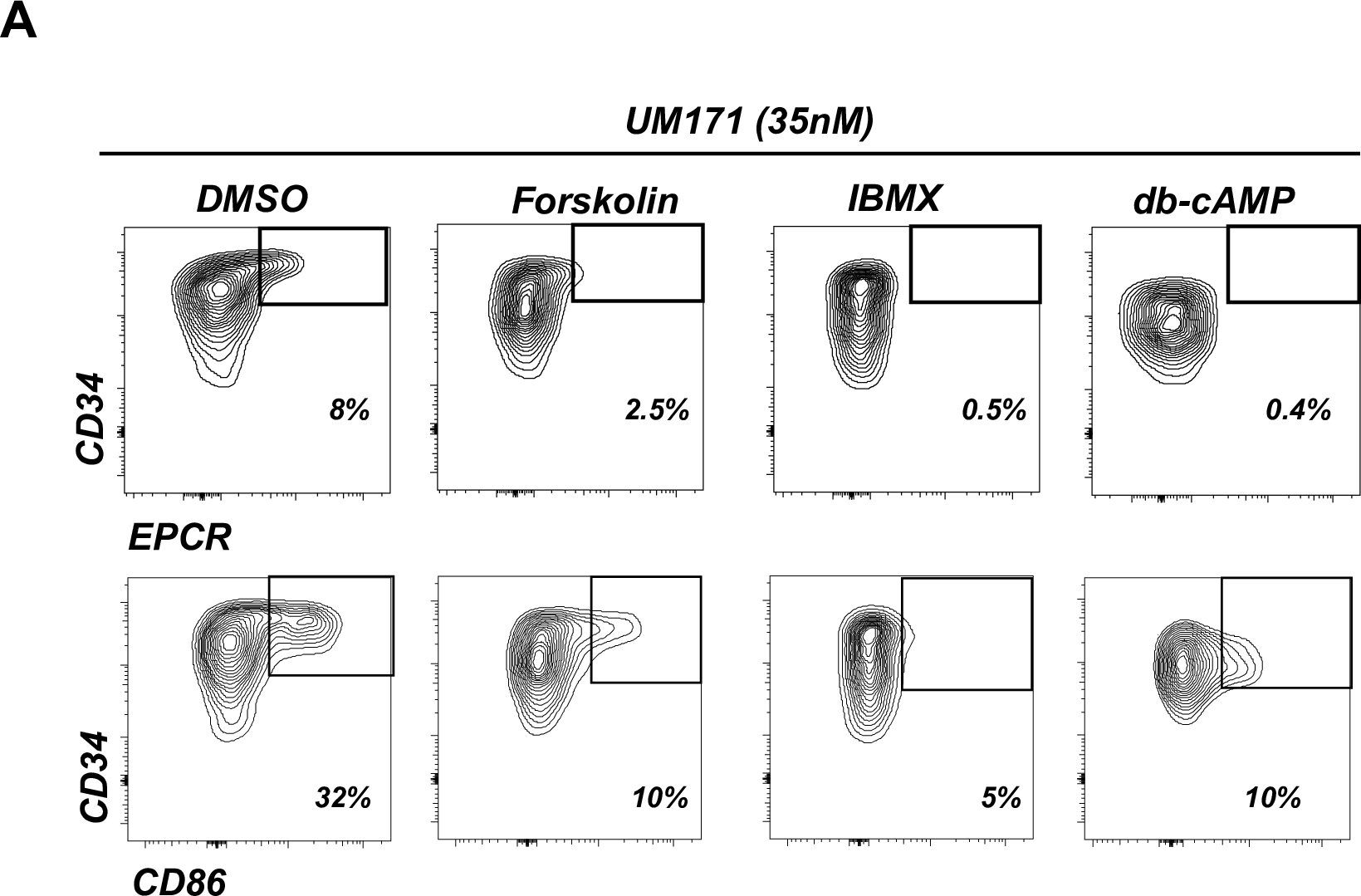

Supplement: S6 Fig — A: CD34+ cord blood cells were exposed to UM171 (35nM) in presence or absence of the indicated cAMP elevating agents (adenylate cyclase activator Forskolin (10microM), phosphodiesterase inhibitor IBMX (200microM) and cell permeable cAMP analog db-cAMP (100M)), cells were then assessed by flow cytometry for UM171 induced expression of expression of CD34, EPCR and CD86. Data show representative FACS profiles of 3 independent experiments. (TIF) [file pone.0224900.s006.tif]

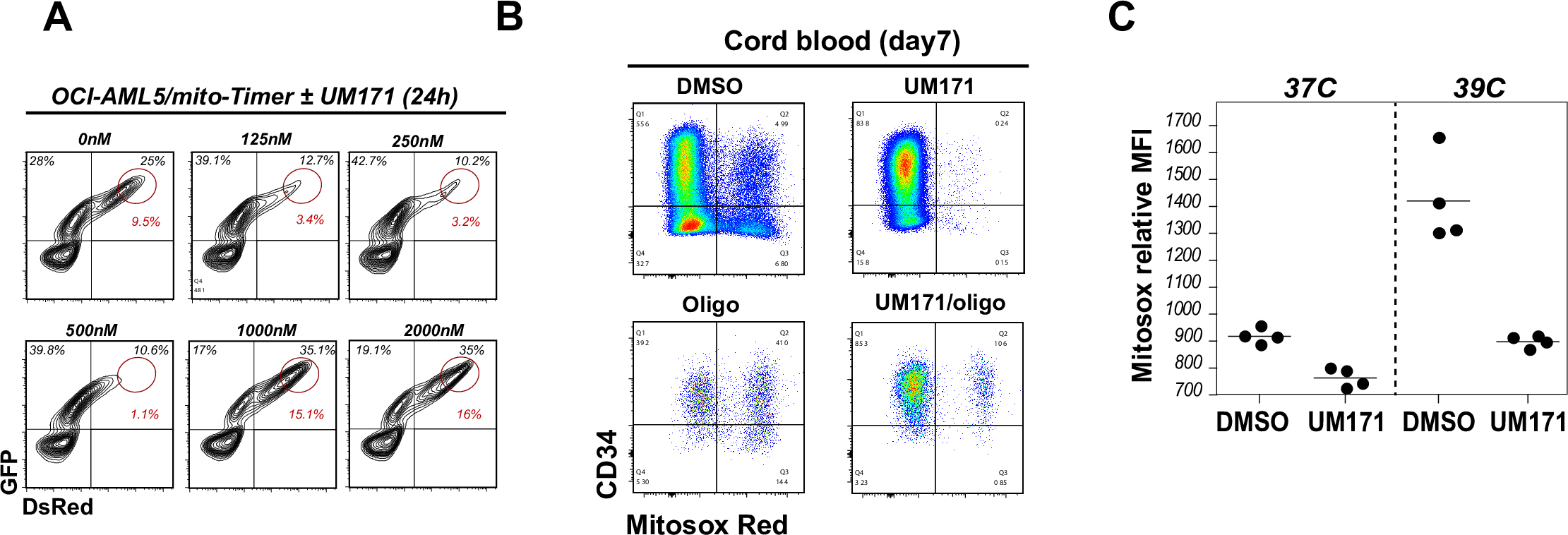

Supplement: S7 Fig — A: OCI-AML5 cells stably expressing Dox-inducible MitoTimer vector were exposed to increasing doses of UM171 for 24hrs and treated with Dox for 3hrs. Cells were the analysed by flow cytometry. Data show representative dot-plot profile of MitoTimer expressing cells (y-axis, green channel; x-axis, red channel). Note that while low dose of UM171 (125 to 500nM) reduces signals in red channel (consistent with lower ROS and improved mitochondrial quality), high dose of UM171 (above 1microM) increase ROS level (enhanced red signal). B: Representative FACS profile of ROS production at day 7 in CD34+ cells exposed or not to UM171± oligomycin. C: CD34+ cord blood cells were exposed for 7 days to DMSO or UM171 (35nM) in mild hyperthermia condition. Mitochondrial ROS were then assessed in HSC enriched CD34+CD45RA- subset by flow cytometry using mitosox staining. (TIF) [file pone.0224900.s007.tif]
