# Supplementary material for: UM171 induces a homeostatic inflammatory-detoxification response supporting human HSC self-renewal
Source: PLoS One. 2019 Nov 8;14(11):e0224900. doi: 10.1371/journal.pone.0224900 (PMC6839847; doi:10.1371/journal.pone.0224900)
Supplement: Attachment — Submitted filename: Response to Reviewers_PONE-D-19-19512.docx [file pone.0224900.s009.docx]

**GENERAL COMMENT**

We would like to thank the reviewers for the time invested in our manuscript and for their constructive comments. As requested, the paper now includes the description of all cell type used in this study. As asked, we also show that NFκB inhibitor affects EPCR at the mRNA levels in HSC enriched subset (new supplementary Figure 5A). We feel that our revised paper fully addresses all of the comments raised by the reviewer and hope this work is now appropriate for publication in Plos One.

***Review Comments to the Author and Responses***


Reviewer #1: Chagraoui et al investigated the roles of UM171 in HSCs and found that UM171 triggers a balanced pro- and anti-inflammatory network that relies on NFKB activation and EPCR-dependent detoxification. This represents a new finding with mechanistic insights. Major comments:

We thank reviewer #1 for his insights and have addressed each of the points raised.

1. Fig 1: The authors may explain why they chose to use OCI-AML5 cell line.

Various myeloid derived cell lines were screened for the upregulation of both EPCR and CD86 in response to UM171 (considered as a signature of UM171 activity in primary hematopoietic cells). Among them, Acute Myeloid Leukemia cell lines OCI-AML3 and OCI-AML5 (FAB M4), promyelocytic leukemia NB4 (FAB M3) and monoblastic leukemia THP-1 (FAB M5) are shown in supplementary Figure 1. OCI-AML5 cell line was chosen for further studies (transcriptome analysis, ROS production, sensitivity to inflammation…) as it shows a high clonogenecity and has the most consistent and highest response to UM171 (based on EPCR and CD86 surface expression).

All informations concerning cell lines are now in Supplementary Figure 1 legend and Materials and Methods sections.

What was the culture condition for this cell line - is it different from that for culture of HSCs?

For all experiments performed in this study, OCI-AML5 cells were cultured in presence of 10% serum and GM-CSF (10ng/ml) according to manufacturer’s. CD34+ cord blood cells were cultured in serum free medium supplemented with SCF, TPO and FLT3L cytokines as we have previously shown that this condition is necessary to achieve optimal HSC expansion and that UM171 cannot compensate for any of these cytokines (Fares I and al, 2014). Unfortunately, culture of OCI-AML5 in serum free medium (as performed for cord blood cells) dramatically affects its survival independently of UM171 presence.

Do medium components affect UM171’s effect on OCI-AML5 cells, which enables the gene expression profiles very different from those of UM171 treated hCB CD34+ cells?

Our work shows that UM171 exhibit inflammatory properties in both OCI-AML5 cells and primitive CD34+ cells and any inflammatory insult will affect UM171 effects. As shown in Figure 3, pro-inflammatory cytokines such as TNFα, exacerbate UM171 effect on both OCI-AML5 and CD34+ cells especially in the absence of EPCR anti-inflammatory properties.

In addition, please include the description of cell type used in Fig 1d-h in the figure legends.

Done

2. Does NFkB inhibitor decrease EPCR mRNA in HSCs?

Yes. NFKB inhibitor (EVP4593) significantly decreased EPCR mRNA levels in HSC enriched CD34+CD45RA- subset. See new supplementary Figure 5A and text.

3. Given that committed cells respond to UM171 differently from primitive cells, the authors may comment on whether UM171 has different effects on hCB CD34+ cells and purified hCB HSCs in terms of stem cell expansion.

This question was addressed in our previous paper “EPCR expression marks UM171-expanded CD34+ cord blood stem cells. Fares I et al, Blood. 2017 Jun 22; 129(25):3344-3351. doi: 10.1182/blood-2016-11-750729. Epub 2017 Apr 13. PMID: 28408459. ” (See main Figure 2).

In this previous study, we have shown that total day3 UM171-expanded cord blood cells and day3 UM171-expanded EPCR+ sorted cells (highly enriched in HSC) exhibit similar in vitro (Figure 2C) and in vivo (Figure 2D) expansion, suggesting that in this setup the effect of UM171 on committed cells (CD34+EPCR-) have little if no impact on HSC subset expansion.

Importantly, using UM171 in combination with a “fed-batch” culture strategy (Csaszar E et al, Cell Stem Cell. 2012 Feb 3; 10(2):218-29. doi: 10.1016/j.stem.2012.01.003. PMID: 22305571) allows the dilution of inhibitory factors produced by committed cells and hence optimal HSC expansion.
